# Association between lipoprotein(a) and peripheral arterial disease in coronary artery bypass grafting patients

**DOI:** 10.1002/clc.24003

**Published:** 2023-03-10

**Authors:** Cheng Yi, Gao Junyi, Liu Fengju, Zhao Qing, Chen Jie

**Affiliations:** ^1^ Department of Diagnostic Ultrasound Beijing Anzhen Hospital affiliated to Capital University of Medical Sciences Beijing China; ^2^ Department of Cardiovascular Medicine Beijing Shijitan Hospital affiliated to Capital University of Medical Sciences Beijing China

**Keywords:** coronary artery bypass grafting (CABG), lipoprotein(a), peripheral arterial disease (PAD)

## Abstract

**Objective:**

Peripheral arterial disease (PAD) affects a large population and is associated with various adverse clinical outcomes. Lipoprotein(a) has proatherogenic properties and is associated with PAD incidence and severity. The aim of this study is to explore the association between LP(a) and PAD in coronary artery bypass grafting (CABG) patients.

**Methods:**

A total of 1001 patients were included and divided into two groups: low Lp(a) group [LP(a) < 30 mg/dL] and high Lp(a) group [LP(a) ≥ 30 mg/dL]. A comparison of PAD incidence diagnosed by ultrasound was made between the groups. Multivariate logistic regression was conducted to explore the risk factors for PAD. During the analysis, the influence of diabetes mellitus (DM) and gender on LP(a) serum level was taken into consideration.

**Results:**

DM history (odds ratio [OR], 2.330, *p* = .000 for males; OR, 2.499, *p* = .002 for females) and age (OR, 1.101, *p* = .000 for males; OR, 1.071, *p* = .001 for females) were risk factors for PAD. LP(a) ≥ 30 mg/dL was a risk factor for PAD only in female patients (OR, 2.589, *p* = .003), while smoking history was a risk factor only in male patients (OR, 1.928, *p* = .000). LP(a) level was not associated with PAD severity in DM patients of both gender. As for female patients without DM, PAD was more severe in the high LP(a) group.

**Conclusions:**

In CABG patients, DM history and age were risk factors for PAD. But a high level of LP(a) was a significant risk factor only in female patients. In addition, we are the first to propose a gender deviation in the correlation between LP(a) serum level and severity of PAD diagnosed by ultrasound.

## Graphical abstract

We proposed gender differences in risk factors for PAD: shown on the left side. Except for traditional risk factors (diabetes mellitus [DM] and age), a high level of LP(a) was a risk factor for PAD but only in female patients and smoking was a risk factor only in male patients. We are the first to propose a gender deviation in the association between LP(a) serum level and PAD severity diagnosed by ultrasound: shown on the right side. A higher incidence of severe PAD was only observed in the high LP(a) level group of female patients without DM.

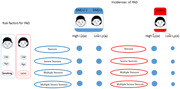

## 1 BACKGROUND

A systematic review estimated that 238 million people were living with peripheral arterial disease (PAD) in 2015 worldwide.^1^ PAD affects a large population and has become an increasingly global problem.^2^ A body of evidence indicated that PAD was associated with an increased risk of various adverse clinical outcomes, thus it should not be underestimated.^1^

Traditionally, the term PAD refers to the disease of vascular beds all over except for the heart, which includes the carotid artery, renal artery, lower extremity artery, and so on. The most common cause of PAD is atherosclerosis followed by peripheral venous disease and lymphatic disease.^3^ There exist different opinions on the definition of PAD in different academic groups.^4^ For the purpose of our research, we referred to PAD as partial or complete atherosclerotic obstruction of the lower extremity artery.^2^

Lipoprotein(a) [LP(a)] is a lipoprotein with a structure similar to low‐density lipoprotein (LDL) synthesized in the liver.^5^ LP(a) serum level is genetically determined. Although its serum level varies among individuals, it is relatively stable within a single individual.^6^ Lp(a) has proinflammatory and proatherogenic properties. Due to these properties, Lp(a) serum level is associated with atherosclerotic cardiovascular diseases including PAD.^7^ Previous studies have shown that Lp (a) is not only a significant independent risk factor for PAD but also associated with more severe forms of PAD in specific populations.^8^, ^9^

The association between LP(a) serum level and PAD in coronary artery bypass grafting (CABG) patients is unknown. We speculated that in this population, LP(a) may have a correlation with PAD. In this study, we aim to explore the risk factors for PAD in CABG patients and the correlation between LP(a) and severity of PAD.

## 2 METHODS

We retrospectively reviewed 1500 consecutive patients undergoing CABG in the Department of Cardiology at Beijing Anzhen Hospital between 2018 and 2020. After excluding patients with incomplete or missing data, 1001 patients were finally included. Patients were divided into two groups: low Lp(a) group [LP(a) < 30 mg/dL] and high Lp(a) group [LP(a) ≥ 30 mg/dL]. We chose 30 mg/dL as the cut‐off value of LP(a) according to research evidence from the Chinese population and previous studies from the Asian population.^8^, ^9^, ^10^ Considering the potential influence of diabetic status and gender on LP(a) serum level, when we discussed the association of LP(a) and severity of PAD, we grouped the patients according to their gender and history of diabetes mellitus (DM).

For all patients, we recorded preoperative baseline characteristics including age, sex, body mass index, DM history, hypertension history, smoking history, systolic blood pressure, diastolic blood pressure, and so on were all recorded. Preoperative biochemical blood tests including serum total cholesterol, triglycerides, high‐density lipoprotein (HDL) cholesterol, LDL cholesterol, Lp(a), and so on were all recorded. Each patient received a preoperative lower extremity artery sonographic examination. Generally, all routine examinations including lower extremity ultrasound were completed within 1 week after admission and then surgery was performed. Four arterial beds were imaged: femoral, popliteal, posterior tibia, and anterior tibia arteries. The femoral artery included common and superficial femoral arteries. All images were acquired with Philips, GE, or Hitachi ultrasound imaging systems by certified experienced ultrasound clinicians. Images were acquired using an L9‐3 transducer. For each artery bed, we define 40%–49% diameter stenosis as mild stenosis, 50%–69% diameter stenosis as moderate stenosis, and 70%–99% diameter stenosis as severe stenosis. Occlusion was grouped into severe stenosis. This study was approved by the Ethics Committee of Beijing Anzhen Hospital.

### 2.1 Statistical analysis

Continuous variables were reported as mean ± standard deviation and categorical variables as numbers and percentages. Univariate comparisons between groups were performed using the *χ* ^2^ test for categorical variables and Student's *t*‐test or Mann–Whitney rank‐sum test for continuous variables, as appropriate. To explore the risk factors for PAD, a multivariate logistic regression analysis was performed. All analyses were performed using SPSS26.0 (SPSS).

## 3 RESULTS

### 3.1 Basic characteristics

A total of 1001 patients were finally included. The mean age was 61.62 ± 8.52 years and 76.8% were male. In the high Lp(a) group, total cholesterol (TCHO) serum level (4.27 ± 1.11 vs. 4.02 ± 1.05, *p* = .001) and LDL serum level (2.69 ± 0.93 vs. 2.39 ± 0.87, *p* = .000) were significantly higher than the low LP(a) group. Triglyceride serum level was slightly higher in the low LP(a) group (1.53 ± 0.83 vs. 1.70 ± 1.28, *p* = .044). The proportion of male patients was similar between the two groups. The proportion of patients with DM history, hypertension (HTN) history, and smoking history were also of no significance between the two groups. The results were shown in Table 1.

**Table 1:** Basic characteristics of patients.

	Total (*N* = 1001)	Low LP(a) group (<30 mg/dL) (*N* = 714)	High LP(a) group (≥30 mg/dL) (*N* = 287)	*p* Value
Age (year)	61.62 ± 8.52	61.66 ± 8.39	61.53 ± 8.85	.826
Male (%)	769 (76.8%)	549 (76.9%)	220 (76.7%)	.936
BMI (kg/m^2^)	25.55 ± 3.19	25.44 ± 3.18	25.82 ± 3.24	.090
History of HTN (%)	636 (63.5%)	458 (64.1%)	178 (62.0%)	.528
History of DM (%)	397 (39.7%)	292 (40.9%)	105 (36.6%)	.207
History of stroke (%)	195 (19.5%)	139 (19.5%)	56 (19.5%)	.987
History of AF (%)	27 (2.7%)	19 (2.7%)	8 (2.8%)	.911
History of MI (%)	247 (24.7%)	174 (24.4%)	73 (25.4%)	.724
Smoking history (%)	499 (49.9%)	357 (50.0%)	142 (49.5%)	.881
Drinking history (%)	292 (29.2%)	213 (29.8%)	79 (27.5%)	.468
Preoperative systolic blood pressure (mm Hg)	130.53 ± 16.33	130.86 ± 16.58	129.71 ± 15.71	.316
Preoperative diastolic blood pressure (mm Hg)	75.89 ± 9.99	75.98 ± 10.01	75.64 ± 9.94	.623
Preoperative heart rate (beat/min)	75.59 ± 10.36	75.60 ± 10.29	75.58 ± 10.53	.980
WBC (G/L)	6.87 ± 1.72	6.84 ± 1.66	6.97 ± 1.83	.254
Alt (mmol/L)	32.44 ± 28.61	32.30 ± 26.56	32.80 ± 33.21	.800
Ast (mmol/L)	27.74 ± 25.52	27.47 ± 24.20	28.43 ± 28.60	.592
CREA (μmol/L)	71.83 ± 14.73	72.05 ± 15.01	71.27 ± 14.01	.449
eGFR (ml/min/1.73 m^2^)	93.02 ± 12.82	92.91 ± 13.03	93.31 ± 12.32	.659
UA (μmol/L)	340.58 ± 86.33	341.99 ± 87.14	337.06 ± 84.33	.414
TG (mmol/L)	1.65 ± 1.17	1.70 ± 1.28	1.53 ± 0.83	**.044**
TCHO (mmol/L)	4.09 ± 1.07	4.02 ± 1.05	4.27 ± 1.11	**.001**
HDL‐C (mmol/L)	1.04 ± 0.25	1.04 ± 0.26	1.03 ± 0.24	.908
LDL‐C (mmol/L)	2.48 ± 0.89	2.39 ± 0.87	2.69 ± 0.93	**.000**
CRP (mg/l)	4.38 ± 6.43	4.14 ± 6.19	4.96 ± 6.99	.070
GA (%)	16.79 ± 4.05	16.91 ± 4.16	16.49 ± 3.78	.142

*Note*: Bold values indicate statistically significant values.

Abbreviations: AF, atrial fibrillation; ALT, alanine aminotransferase; AST, aspartate aminotransferase; BMI, body mass index; CREA, creatinine; CRP, C‐reactive protein; DM, diabetes mellitus; eGFR, estimated glomerular filtration rate; HDL, high‐density lipoprotein; HTN, hypertension; LDL, low‐density lipoprotein; MI, myocardial infarction; TCHO, total cholesterol; TG, triglyceride; UA, uric acid; WBC, white blood cell.

### 3.2 Incidence of PAD in CABG patients

The incidence of PAD was 47.0% (48.9% in male patients and 40.5% in female patients). The incidence of PAD had a significant difference between males and females (*p* = .025).

### 3.3 Correlation between LP(a) serum level and PAD severity

The results were shown in Tables 2 and 3. As for DM patients, there was no significant correlation between LP(a) serum level and severity of PAD for nearly all artery beds (anterior tibial artery, posterior tibial artery, and the entire lower extremity artery) in male and female patients. In male patients, incidences of PAD in some arterial beds were slightly higher in the low LP(a) group, although there might be no significant difference. In addition, the low LP(a) group had a higher incidence of femoral popliteal artery stenosis (34.4% vs. 20.3%, *p* = .027) and multiple vessel femoral popliteal artery stenosis (19.6% vs. 4.3%, *p* = .002). In female patients, the incidence of PAD in the high and low LP(a) groups were of no significant difference.

**Table 2:** Correlation between LP(a) and PAD severity in two groups (patients with DM).

	Male (*N* = 293)			Female (*N* = 104)		
	Low LP(a) group (*N* = 224)	High LP(a) group (*N* = 69)	*p* Value	Low LP(a) group (*N* = 68)	High LP(a) group (*N* = 36)	*p* Value
Lower extremity artery stenosis (%)	137 (61.2%)	36 (52.2%)	.184	33 (48.5%)	22 (61.1%)	.221
Severe lower extremity artery stenosis (%)	98 (43.8%)	26 (37.7%)	.372	26 (38.2%)	15 (41.7%)	.733
Multiple vessel lower extremity artery stenosis (%)	106 (47.3%)	27 (39.1%)	.232	23 (33.8%)	13 (36.1%)	.816
Multiple vessel severe lower extremity artery stenosis (%)	63 (28.1%)	20 (29.0%)	.890	18 (26.5%)	8 (22.2%)	.634
Femoral popliteal artery stenosis (%)	77 (34.4%)	14 (20.3%)	**.027**	15 (22.1%)	14 (38.9%)	.069
Severe femoral popliteal artery stenosis (%)	16 (7.1%)	2 (2.9%)	.319	3 (4.4%)	2 (5.6%)	.797
Multiple vessel femoral popliteal artery stenosis (%)	44 (19.6%)	3 (4.3%)	**.002**	11 (16.2%)	9 (25.0%)	.277
Multiple vessel severe femoral popliteal artery stenosis (%)	3 (1.3%)	0 (0%)	.778	1 (1.5%)	1 (2.8%)	.652
Anterior or posterior tibial artery stenosis (%)	110 (49.1%)	31 (44.9%)	.543	29 (42.6%)	17 (47.2%)	.655
Severe anterior or posterior tibial artery stenosis (%)	98 (43.8%)	26 (37.7%)	.372	26 (38.2%)	15 (41.7%)	.733
Multiple vessel anterior or posterior tibial artery stenosis (%)	86 (38.4%)	26 (37.7%)	.915	20 (29.4%)	9 (25.0%)	.633
Multiple vessel severe anterior or posterior tibial artery stenosis (%)	61 (27.2%)	19 (27.5%)	.960	17 (25.0%)	8 (22.2%)	.752

*Note*: Bold values indicate statistically significant values.

Abbreviations: DM, diabetes mellitus; PAD, peripheral arterial disease.

**Table 3:** Correlation between LP(a) and PAD severity in two groups (patients without DM).

	Male (*N* = 476)			Female (*N* = 128)		
	Low LP(a) group (*N*=325)	High LP(a) group (*N* = 151)	*p* Value	Low LP(a) group (*N* = 97)	High LP(a) group (*N* = 31)	*p* Value
Lower extremity artery stenosis (%)	134 (41.2%)	69 (45.7%)	.359	24 (24.7%)	15 (48.4%)	**.013**
Severe lower extremity artery stenosis (%)	91 (28.0%)	50 (33.1%)	.256	15 (15.5%)	11 (35.5%)	**.016**
Multiple vessel lower extremity artery stenosis (%)	96 (29.5%)	54 (35.8%)	.174	17 (17.5%)	13 (41.9%)	**.005**
Multiple vessel severe lower extremity artery stenosis (%)	55 (16.9%)	34 (22.5%)	.145	7 (7.2%)	6 (19.4%)	.108
Femoral popliteal artery stenosis (%)	65 (20.0%)	37 (24.5%)	.265	11 (11.3%)	11 (35.5%)	**.002**
Severe femoral popliteal artery stenosis (%)	12 (3.7%)	6 (4.0%)	.881	1 (1.0%)	3 (9.7%)	.069
Multiple vessel femoral popliteal artery stenosis (%)	33 (10.2%)	22 (14.6%)	.161	6 (6.2%)	9 (29.0%)	**.002**
Multiple vessel severe femoral popliteal artery stenosis (%)	4 (1.2%)	3 (2.0%)	.819	0 (0%)	2 (6.5%)	.091
Anterior or posterior tibial artery stenosis (%)	107 (32.9%)	60 (39.7%)	.147	18 (18.6%)	11 (35.5%)	**.050**
Severe anterior or posterior tibial artery stenosis (%)	91 (28.0%)	50 (33.1%)	.256	15 (15.5%)	11 (35.5%)	**.016**
Multiple vessel anterior or posterior tibial artery stenosis (%)	77 (23.7%)	42 (27.8%)	.334	12 (12.4%)	6 (19.4%)	.498
Multiple vessel severe anterior or posterior tibial artery stenosis (%)	49 (15.1%)	34 (22.5%)	**.046**	7 (7.2%)	5 (16.1%)	.259

*Note*: Bold values indicate statistically significant values.

Abbreviations: DM, diabetes mellitus; PAD, peripheral arterial disease.

As for patients without DM, there was also no significant correlation between LP(a) serum level and severity of PAD of almost all artery beds in male patients except for multiple vessel severe anterior or posterior tibial artery stenosis [22.5% in the high LP(a) group and 15.1% in the low LP(a) group, *p* = .046]. In female patients, the situation was different. Compared to the low LP(a) group, the incidence of lower extremity artery stenosis (48.4% vs. 24.7%, *p* = .013), severe lower extremity artery stenosis (35.5% vs. 15.5%, *p* = .016), multiple vessel lower extremity artery stenosis (41.9% vs. 17.5%, *p* = .005), femoral popliteal artery stenosis (35.5% vs. 11.3%, *p* = .002), multiple vessel femoral popliteal artery stenosis (29.0% vs. 6.2%, *p* = .002), anterior or posterior tibial artery stenosis (35.5% vs. 18.6%, *p* = .005), and severe anterior or posterior tibial artery stenosis (35.5% vs. 15.5%, *p* = .016) were all significantly higher in the high LP(a) group. Although the sample in this group was small and the conclusion might be put forward conservatively, we could still see the trends.

### 3.4 Risk factors for PAD

Our results showed that in CABG patients, risk factors for PAD had obvious gender differences. Surprisingly, LDL was not an independent risk factor in both male and female patients.

In male patients, risk factors for PAD included smoking history [odds ratio (OR), 1.928, 95% CI (1.387–2.679), *p* = .000], DM history [OR, 2.330, 95% CI (1.684–3.224), *p* = .000] and age [OR, 1.101, 95% CI (1.079–1.125), *p* = .000]. LP(a) was not a risk factor for PAD in male patients (Table 4).

**Table 4:** Multivariate logistics analysis of risk factors for PAD (male).

	OR (95% CI)	*p* Value
Age	1.101 (1.079–1.125)	.000
Smoking history	1.928 (1.387–2.679)	.000
DM	2.330 (1.684–3.224)	.000
LP(a) ≥ 30 mg/dL	0.965 (0.682–1.364)	.838
LDL	1.201 (0.999–1.444)	.051

*Note*: Bold values indicate statistically significant values.

Abbreviations: CI, confidence interval; DM, diabetes mellitus; LDL, low‐density lipoprotein; OR, odds ratio; PAD, peripheral arterial disease.

As for female patients, LP(a) emerged as an independent risk factor. For female patients, LP(a) ≥30 mg/dL had a nearly 2.6 times risk of PAD [OR, 2.589, 95% CI (1.376–4.870), *p* = .004]. History of DM [OR, 2.499, 95% CI (1.411–4.427), *p* = .002] and age [OR, 1.071, 95% CI (1.027–1.118), *p* = .001] was still independent risk factors for PAD (Table 5).

**Table 5:** Multivariate logistics analysis of risk factors for PAD (female).

	OR (95% CI)	*p* Value
Age	1.071 (1.027–1.118)	.001
Smoking history	0.888 (0.339–2.327)	.810
DM	2.499 (1.411–4.427)	.002
LP(a)≥30 mg/dL	2.589 (1.376–4.870)	.003
LDL	1.132 (0.842–1.522)	.441

Abbreviations: CI, confidence interval; DM, diabetes mellitus; LDL, low‐density lipoprotein; OR, odds ratio; PAD, peripheral arterial disease.

## 4 DISCUSSION

In CABG patients, the incidence of PAD was 47.0%. In male patients, the incidence of PAD was 48.9%, and in female patients, it was 40.5%. Our study showed that the incidence of PAD in CABG patients was quite high which was quite reasonable. Atherosclerosis is a systemic disease and affects multiple vascular beds.^11^ PAD is always coexistent with coronary artery disease (CAD).^12^ The significance of our study was that we provided the incidence of PAD in a CABG population and we used the ultrasonic method to evaluate PAD severity. In previous studies, PAD was always diagnosed using the ankle‐brachial index (ABI), which is easier to perform but still has shortcomings. For instance, ABI can be falsely high in the presence of artery calcification, a condition often observed in patients with severe CAD such as CABG.^13^ The ultrasonic method was cheap, safe, and had a satisfying accuracy as well. The sensitivities of ultrasound in detecting occlusion and stenosis have been reported to be 95% and 92% and specificities were 99% and 97%, respectively.^14^ Due to these advantages, the application rate of lower extremity artery ultrasound in our hospital is quite high, and nearly all CABG patients underwent this examination. We propose that ultrasound might be a more suitable method for PAD at least in our country. First, ultrasound is more intuitive and we can directly see the lumen of any segment of the artery. The degree of stenosis can be accurately measured in any segment of the artery. A skilled ultrasonic physician can complete the exam quickly and accurately. Second, from the perspective of follow‐up, the ultrasound method might be more suitable. Since ultrasound could record stenosis location and progression more accurately and intuitively, we could monitor the progress of the disease in future follow‐ups.

PAD has been traditionally considered as affecting men more than women and some scholars ascribed this to hormonal differences.^15^, ^16^ But this point of view was controversial. Researchers also proposed that women had an equal or even higher prevalence of PAD diagnosed using ABI measurements.^3^, ^17^ In our study, the incidence of PAD (48.9% vs. 40.5%) was higher in male patients.

According to previous studies, DM history, HTN history, smoking history, dyslipidemia, and sedentary lifestyle are conventional risk factors for PAD,^18^, ^19^, ^20^ while C‐reactive protein (CRP) and LP(a) are inflammatory risk factors for PAD.^19^ We conclude that in CABG patients, DM history, age, and smoking history were independent risk factors for PAD in male patients. The result was consistent with the previous findings. For female patients, smoking history was no longer a risk factor and LP(a) emerged as a risk factor. Our results indicated that for female patients undergoing CABG, physicians always concentrated on traditional risk factors such as LDL (which we will discuss later), but more attention should be paid to the serum level of LP(a). For male patients, physicians should pay more attention to unhealthy habits such as smoking.

To our surprise, LDL was not an independent risk factor. LDL was a conventional risk factor for PAD as well as other cardiovascular diseases.^21^, ^22^ We speculate the possible reasons might be the influence of certain drugs. Since CABG patients were always with multiple comorbidities such as hyperlipidemia. The application of these drugs must affect LDL serum level, which we can clearly see from the basic characteristics that the mean serum level of LDL was 2.47 ± 0.899 mmol/L. Thus, the predictive value of LDL may have been concealed, while LP(a) level emerged as an important surrogate.

The association between elevated LP(a) serum level and PAD has already been proposed.^23^ Prior studies have also reported an association between increased Lp(a) serum level and PAD severity. Early in 1997, Hong Kong researchers studied 200 PAD patients with clinical symptoms and found that patients with a higher LP(a) serum level had more severe clinical symptoms, which included resting pain and ulcerations.^8^ In 2020, Yanaka et al.^9^ studied 108 Japanese patients who underwent endovascular therapy for lower extremity atherosclerosis lesions and found that the incidence of severe angiographic results of femoropopliteal lesions was higher in patients with higher LP(a) serum level.

Unlike previous studies, we found the phenomenon of gender deviation. The correlation between LP(a) serum level and PAD severity was not significant in male patients with or without DM. Since male patients accounted for the majority of the population (76.8%), the level of LP(a) could not add any further value in regard to PAD in a majority of this population. But we still think this result could be a useful supplement for the research field of association between LP(a) and PAD. In addition, ours was a cross‐sectional study. We speculate that LP(a) might be related to the prognosis or progression of male PAD.

As for female patients, we have to admit that the sample size of female patients with high LP(a) levels was small, thus the result might not be so convincing. But the incidence of severe stenosis of different segments of the lower extremity artery in the high LP(a) level group is much higher, thus we believe that this trend is true, and a still larger sample size is needed to obtain a more convincing result.

Although the correlation between LP(a) level and PAD severity in non‐DM female patients needs to be put forward conservatively, we might still see the trend. Surprisingly, the trend was quite reasonable and there might be some possible explanations. LP(a) was recognized as an independent risk factor for CAD, stroke, PAD, and so on, but the situation might be somewhat more complex in women.^24^, ^25^ Although LP(a) serum level remains relatively stable in an individual's life, it may change in some specific circumstances. For instance, LP(a) serum level increases by about 25% after the onset of menopause.^26^ In our study, most female patients were in postmenopausal status. After menopause, LP(a) serum level increases; while the protection of estrogen fades, the incidence of cardiovascular disease then gradually increases. Thus, we speculate that the increase of LP(a) serum level might promote the aggravation of PAD [we can not say that increase of LP(a) serum level directly leads to PAD]. Moreover, as part of systemic vascular disease, lower extremity artery disease may only be one of the manifestations. It is possible that an increase in LP(a) serum level and worsening of PAD occur in parallel.

In addition, LP(a)'s function and atherogenicity might be modified by glycation in the background of diabetes. Rasouli and Mohseni Kiasari^27^ proposed that LP(a) may act synergistically in the presence of diabetes in predicting the severity of CAD. Our result indicated that the correlation between LP(a) serum level and PAD severity was not observed in patients with DM history. Surprisingly, researchers have proposed that DM patients always have reduced Lp(a) levels.^28^ We speculated that the existence of DM might have some influence on the function of LP(a), thus concealing the relation between LP(a) serum level and PAD. As our results had shown in male patients with DM incidence of PAD of several arterial beds was even higher in the low LP(a) group.

In short, our results confirmed previous arguments that LP(a) was more of a “conditional risk factor” and its correlation with PAD as well as other clinical conditions depends on an individual's background based on age, gender, and coexisting conditions such as DM.^29^ As far as we know, we are the first to explore this correlation using the diagnostic method of ultrasound. There is another innovative part that we found, which is the gender deviation of this correlation in a certain population. For male CABG patients with or without DM, high LP(a) serum level was not associated with the severity of PAD. For female CABG patients with DM, this correlation was not significant as well. For female patients without DM, in the high LP(a) level group, a higher incidence of severe PAD was observed. We speculate that in female patients, especially postmenopausal females, LP(a) might have some prognostic effect and lowering LP(a) level might improve the prognosis of PAD.^30^

## 5 CONCLUSION

The incidence of PAD was higher in CABG patients than that in the general population. Unlike the previous studies, we proposed gender differences in the risk evaluation of PAD. Except for traditional risk factors, LP(a) ≥ 30 mg/dL was a risk factor for PAD, but only for female patients. In addition, we are the first to propose a gender deviation in the association between LP(a) serum level and PAD severity diagnosed by ultrasound. A higher incidence of severe PAD was observed only in the high LP(a) level group of female patients without DM.

## 6 LIMITATION

First, the sample in non‐DM females with high LP(a) levels was quite small. There were certain reasons: ① women accounted for only a small proportion of CABG patients (23.2%), ② the number of women without diabetes is also small, and ③ because LP(a) serum levels showed a skewed distribution, there are fewer patients with more than 30 mg/dL level. Under this condition, the proportion of female non‐DM patients with a high LP(a) serum level in the total sample was small. Although the incidences of stenosis of different segments in the high LP(a) level group were much higher and the trend was reasonable, a larger sample size is still needed to obtain a more convincing result. Second, our study was only a cross‐sectional study. If we follow up for a period of time and observe the progression of PAD, we might find some impact of LP(a) on the prognosis of PAD. In addition, we could further study whether lowering LP(a) level improves the prognosis of PAD. Third, Lp(a) serum level is affected by certain lipid‐lowering medications. In the CABG population, some patients were diagnosed with hyperlipidemia and took lipid‐lowering drugs. The effect of these drugs on the serum level of Lp(a) could not be calculated.

## CONFLICT OF INTEREST STATEMENT

The authors declare no conflict of interest.

## Data Availability

All data included in this study are available upon request by contacting the corresponding author.
